# Gyroscopic Radiosurgery for the Treatment of Trigeminal Neuralgia

**DOI:** 10.7759/cureus.81011

**Published:** 2025-03-22

**Authors:** Morena Sallabanda, Kita Sallabanda

**Affiliations:** 1 Radiation Oncology, Institute of Advanced Radiosurgery (IRCA), Madrid, ESP; 2 Neurosurgery, Institute of Advanced Radiosurgery (IRCA), Madrid, ESP

**Keywords:** gyroscopic radiosurgery, pain, radiomodulation, trigeminal neuralgia, zap-x

## Abstract

Introduction: Radiosurgical treatment of trigeminal neuralgia (TN) is the most common functional radiosurgery procedure used to modulate refractory pain. The aim of this study is to describe the clinical results and dosimetric parameters of the first series of patients with TN treated with ZAP-X gyroscopic frameless radiosurgery (ZAP Surgical Systems, Inc., San Carlos, CA), an innovative, frameless radiosurgical device.

Methods: A total of 30 patients with TN received gyroscopic frameless radiosurgery (GRS) between February 2023 and January 2025. Treatment plans were developed delivering a maximum dose of 90 Gy in a target that covered a 5-mm segment of the trigeminal nerve in the retrogasserian location using a single isocenter (5 mm collimator). Clinical and treatment information were analyzed with a specific focus on demographic characteristics, etiology, previous treatments, Barrow Institute (BNI) Pain Intensity Scale before and after GRS (pain relief response), the time to pain relief, and the rate of complications and recurrences.

Results: 30 patients (21 females and 9 males) with a median age of 66 years (range: 31-92 years) underwent GRS for refractory TN. The median time from first diagnosis to GRS was 4 years (range: 6 months to 18 years). All patients had received a first therapeutic pharmacological line, 15 patients received at least a radiofrequency thermocoagulation, 7 patients received nerve blocks, 4 patients were treated with botox injection and 2 patients had previous radiosurgery. The median BNI Pain Intensity Scale before GRS was IV (range IIIa-V). The median treatment time was 37 minutes using a median of 221 beams. Median V12 and V10 of the brainstem were 0.04cc (range: 0-0.16cc) and 0.07cc (range: 0-0.24cc), respectively. The median BNI Pain Intensity Scale after GRS was II (range: I-VI) with 80% of patients reporting no pain or occasional pain without medication. The median time to pain relief was 15 days (range: 1-60 days). Five patients (16.6%) presented ipsilateral facial hypesthesia after GRS. 10% of patients presented with recurrent pain 2-6 months after GRS.

Conclusions: GRS is a precise tool for treating TN, demonstrating safety and effectiveness in what is, to our knowledge, the first reported case series documenting its use.

## Introduction

Trigeminal neuralgia (TN) is a severe facial pain diagnosed in a clinical setting. Symptoms are described as unilateral, electric-like, lancinating pain with an abrupt onset and spontaneous remission without objective neurological deficit. TN can be caused by a tumor, multiple sclerosis, or skull-base structural abnormalities such as vascular compression. Idiopathic TN refers to all causes without an established etiology. The first therapeutic line is pharmacological. Nonetheless, refractory TN can become a multidisciplinary challenge [1,2].

When radiation is focally delivered to brain tissue at sub-ablative doses, neural activity may be altered. When done at a specific brain circuit node or connection, this is referred to as radiomodulation. Régis et al. proposed that stereotactic radiosurgery (SRS) could produce a non-ablative modulation effect in different pathologies, consistently showing a clinically positive effect either in pain management, epilepsy, or tremor symptoms immediately following treatment or shortly after, without generating a permanent lesion in the neuronal circuitry [2-4].

Stereotactic radiosurgery (SRS) of the trigeminal nerve is widely used to treat refractory facial pain. This treatment is characterized by the delivery of high doses (60 to 97 Gy) in a specific target, usually as the nerve courses the prepontine cistern. A systematic review of the literature focused on the outcomes of SRS for TN found 65 studies reporting on 6,461 patients with a median of freedom from pain (FFP) response with or without medication of 79% or higher. While most of the reported cases were treated with frame-based SRS, mask-based, image-guided SRS using linear accelerators (LINAC) have also been demonstrated as safe, effective, and less invasive treatment options for TN. The Barrow Neurological Institute (BNI) Pain intensity scale (I: no pain; II: occasional pain, not requiring medication; III: some pain, controlled with medication; IV: some pain, not controlled with medication; V: severe pain/no pain relief) is the most extensively used classification to report outcomes after SRS [1,5].

ZAP-X® (ZAP Surgical Systems, Inc., San Carlos, CA) is a new, image-guided LINAC-based radiosurgery system dedicated to the treatment of intracranial and cervical spine lesions. This system has a 3.0 megavolt (MV) S-band LINAC mounted within a combination of yoked gimbals that accurately rotate around a common isocenter, performing a gyroscopic irradiation. Conical collimators ranging from 4 to 25 mm in diameter are used for beam shaping. It is “self-shielded” with nearly all radiation being contained within the device and allowing it to be used safely without a radiation therapy vault [3,6,7].

The aim of this study is to describe the clinical results of the first series of patients with refractory TN treated with ZAP-X gyroscopic frameless radiosurgery (GRS), an innovative, SRS-dedicated technology, specifically designed to precisely deliver high doses to millimetric intracranial targets.

## Materials and methods

A series of 30 patients with TN received GRS between February 2023 and January 2025. All patients with refractory TN were considered candidates regardless of the etiology. Information about age, gender, date of diagnosis, etiology, previous treatments, BNI Pain Intensity Scale before and after GRS (pain relief response), the time to pain relief, and the rate of complications and recurrences were collected in a database and analysed.

A maximum dose of 90 Gy (prescribed to the 100% isodose line) was delivered inside a target that covered a 5-mm segment of the trigeminal nerve in the retrogasserian location using a single isocenter. A V70 ≥ 50% was considered mandatory for the target volume. Although ZAP-X has a 4 mm collimator, the 5 mm collimator was selected for the treatment of this first series to ensure that the entire target, the thickness of the nerve, and the nerve motion uncertainties were covered). Healthy brain, optic pathway, brainstem, ipsilateral temporal lobe, and cochleas were contoured as organs at risk (OARs), and dose restrictions were applied following international guidelines. For the brainstem, V12 <0.2 cc and V10<0.5 cc were prioritized in order to avoid brainstem necrosis [8,9].

**Figure 1:**
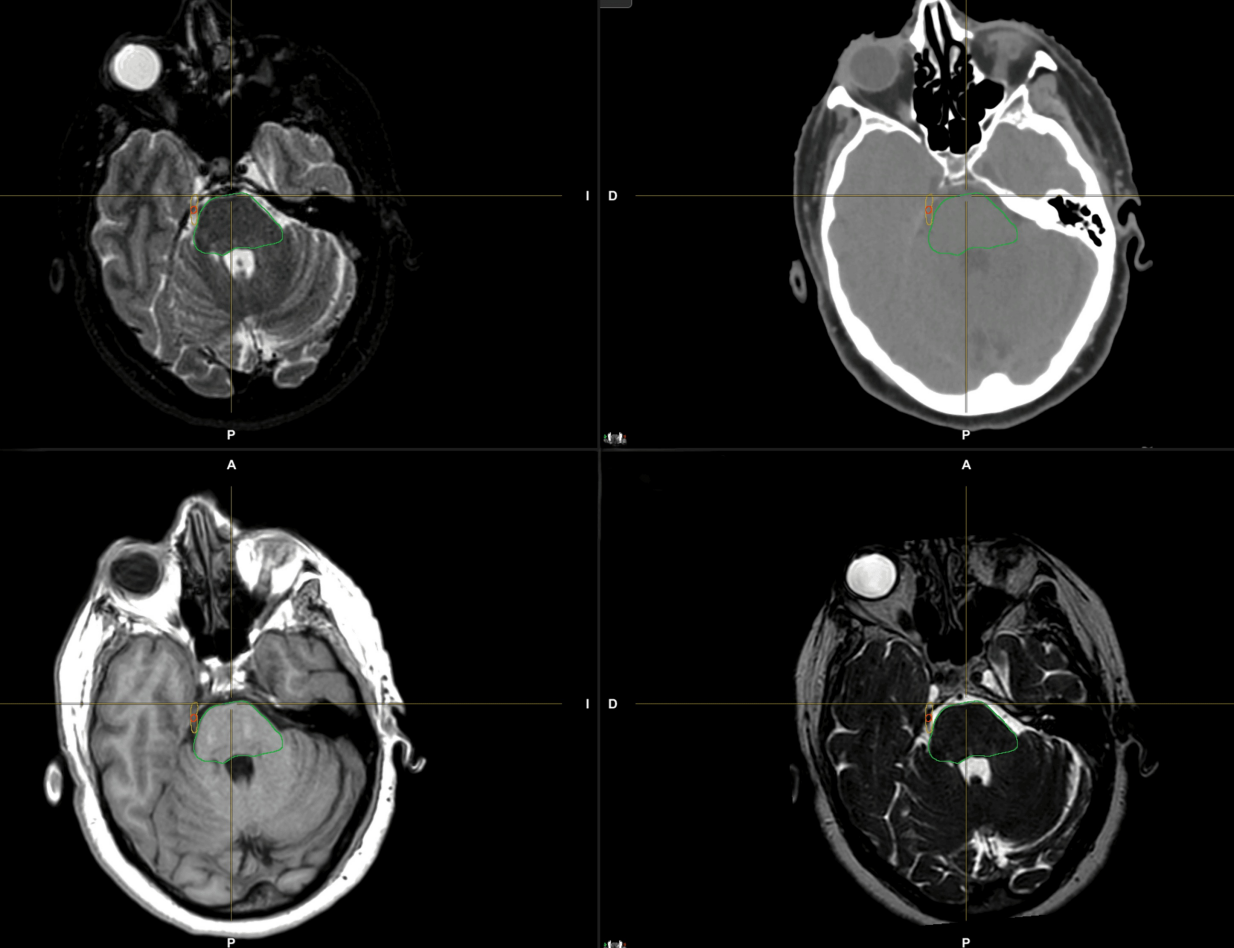
Target volume delineation in the right trigeminal nerve.

 An example of target volume delineation is shown in Figure 1.

For volume delineation, a computerized tomography (CT) and 1.5 Tesla magnetic resonance imaging (MRI) 3D T1, T2, and FIESTA sequences were acquired without contrast in the supine position. The slice thickness was selected to be lower or equal to 1 mm for both images, so enough spatial resolution is guaranteed. A thermoplastic mask was used for immobilization. Rigid and deformable registration together with contouring was performed in Brainlab Elements Software (Brainlab AG, Munich, Germany), using all the sequences to correctly identify the entire extension of the trigeminal nerve and to better determine the target volume.

For treatment planning, the ZAP-X GRS platform works with a dedicated system called ZAP-X TPS which is integrated with the ZAP-X delivery system. The dose calculation was based on the ray-tracing algorithm. A combination of forward-planning techniques to manually placed shots and an inverse planning optimization is used to obtain an optimal solution regarding beam arrangement. The treatment plans were reviewed by a radiation oncologist, a neurosurgeon, and a medical physicist who evaluated the isodose distribution and reviewed the dose-volume histogram (DVH) of the target volume and OARs [10]. 

Target localization is achieved by an integrated planar kilovolt (kV) imaging system providing 3D patient registration. Automatic realignment is performed prior to and throughout the treatment allowing for a frameless setup and tracking. The dose rate is 1500 MUs per minute. In addition, the utilization of very low energy and a very short source-to-axis distance (SAD) of 45 cm optimizes a rapid peripheral dose fall-off and a steep dose gradient. Furthermore, a dose monitor ionization chamber measures the amount of real-time radiation delivered to ensure treatment accuracy [3,6,7].

**Figure 2:**
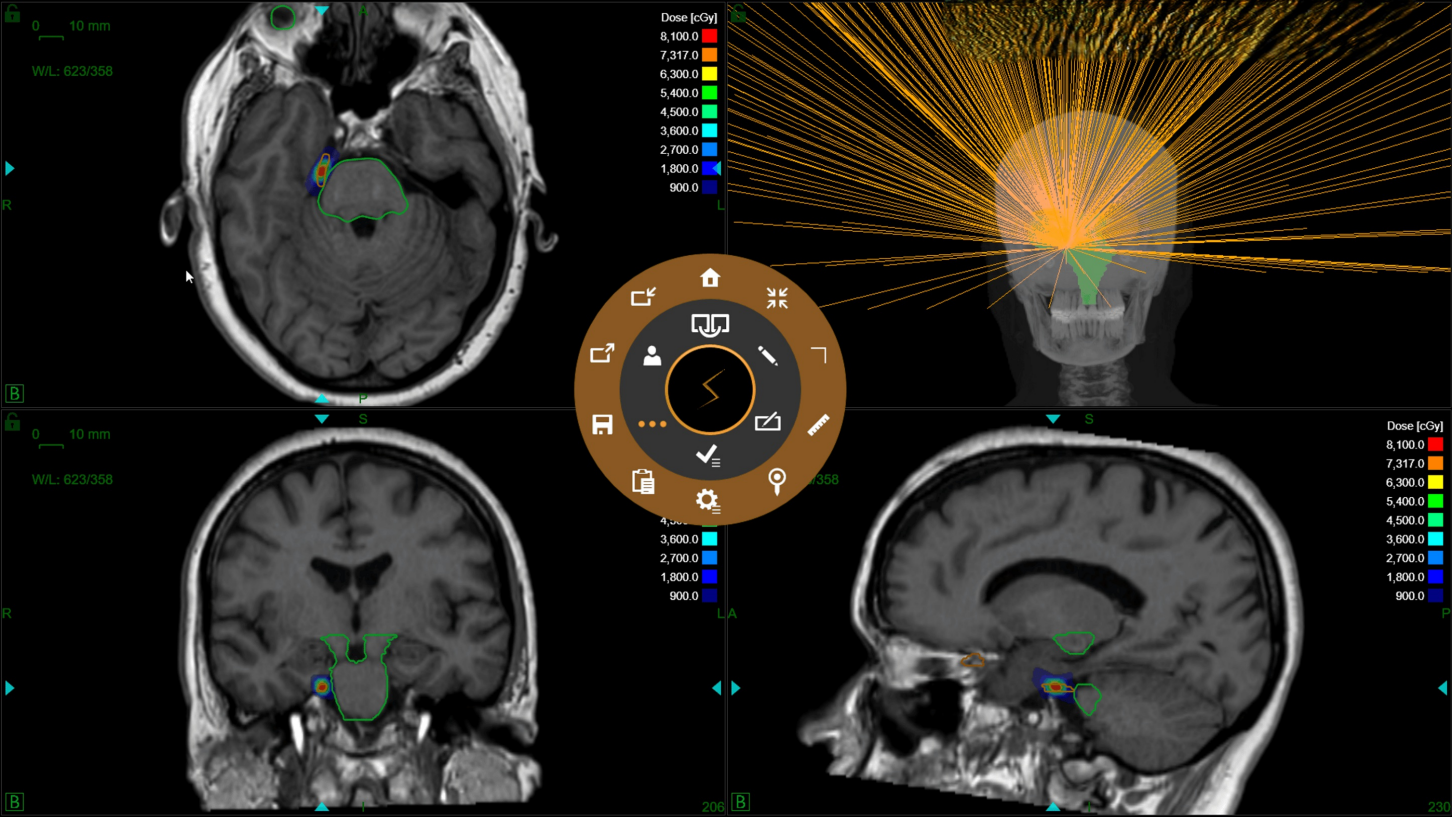
Dose distribution and beam evaluation in a patient with right trigeminal neuralgia.

An example of isodose distribution is shown in Figure 2.

The first clinical follow-up was scheduled two weeks after treatment and, depending on the pain relief response, a personalized medication reduction protocol was performed. BNI Pain Intensity Scale and relevant toxicities were evaluated on each visit. Patients were asked about symptoms such as hypesthesia, dry eye, corneal anesthesia, or diesthesia.

A descriptive analysis of the collected data was performed, creating frequency tables on qualitative variables and describing the median and range of quantitative variables.

## Results

A total of 30 patients (21 females and 9 males) with a median age of 66 years (range: 31-92 years) underwent GRS for refractory TN. Regarding etiology, half of the series (15 patients) were classified as idiopathic, without describing any known cause related to pain onset. On the other hand, 6 patients developed TN after a dental procedure (either extraction or manipulation), 4 patients' TN was related to benign tumors with nerve compression (meningiomas), 3 patients had multiple sclerosis, one patient developed TN after a meloplasty and one patient after trigeminal herpes zoster.

The median time from first diagnosis to GRS was 4 years (range: 6 months to 18 years). All patients had received a first therapeutic pharmacological line, 15 patients received at least a radiofrequency thermocoagulation, seven patients received nerve blocks, four patients were treated with botox injection and two patients had previous radiosurgery. The median BNI Pain Intensity Scale before GRS was IV (range: IIIa-V).

Regarding treatment characteristics, the median treatment time was 37 minutes (range: 31-45 minutes) using a median of 221 beams (range: 98-395 beams). The high variability regarding treatment time and number of beams depended on the number of paths used for beam arrangement during treatment planning (6-17 paths). Since the anatomy of the nerve and the relationship with the brainstem was variable between patients, the shorter the distance of the target with the brainstem, the higher the number of paths and beams needed in order to optimally shape dose distribution, optimizing target coverage and dose gradient around the brainstem. Median V12 and V10 of the brainstem were 0.04cc (range: 0-0.16cc) and 0.07cc (range: 0-0.24cc), respectively. Median V70 of the target volume was 87% (range: 62-100%). Median D0.03cc to the ipsilateral temporal lobe was 13 Gy (range: 5-25 Gy). The median mean dose to the trigeminal nerve was 39 Gy (range: 34 - 52 Gy).

With a median follow-up of eight months (range: 2-24 months), 100% of the patients showed at least some improvement after GRS. The median BNI Pain Intensity Scale after GRS was II (range: I-VI) with 80% of patients reporting no pain or occasional pain without medication. The median time to pain relief was 15 days (range: 1-60 days). Pain recurrence was present in 3 patients (10%), 2-6 months after the first GRS. Two of them were eligible candidates for a second GRS, since there was a six-month interval between the first treatment, both patients showed at least one scale of improvement after the second GRS.

Complications after treatment were mild; five patients (16.6%) presented ipsilateral facial hypesthesia after GRS (of these, two had a previous SRS and one patient already had a mild hypesthesia before the treatment).

## Discussion

Trigeminal neuralgia (TN) remains one of the most challenging pain conditions to manage, particularly in refractory cases. The study presented here examines the first series of patients treated with ZAP-X® GRS.

The patient cohort included a variety of TN etiologies. This broad representation of TN subtypes strengthens the generalizability of the results, indicating that GRS can be beneficial for patients with diverse underlying causes of TN. Most patients in this series had received prior pharmacological treatments or invasive therapies, such as radiofrequency thermocoagulation or nerve blocks, emphasizing the refractory nature of their condition. The median BNI Pain Intensity Scale before treatment was IV, reflecting the severity of pain these patients experienced prior to GRS, and most patients reported significant improvement in pain levels following the treatment.

The median BNI Pain Intensity Scale score after GRS improved to II, demonstrating that a substantial portion of patients experienced significant relief. Although a longer follow-up is necessary to further validate these results, the high rate and median time to pain relief is consistent with previous reports on SRS for TN. Near-immediate pain relief in a subset of patients cannot be related to cellular changes that require several months to manifest. This observation suggests that SRS may modulate neuronal transmission in grossly intact neurons [1,4,10-14].

The precise delivery of radiation is a hallmark of the ZAP-X system. The treatment planning process with ZAP-X involved careful attention to dose distribution. This is particularly important in the context of TN, where the goal is to achieve sufficient radiation to modulate the trigeminal nerve while avoiding damage to critical structures such as the brainstem. The 5-mm collimator was chosen in this first series over the 4-mm collimator in order to ensure adequate coverage and, despite the larger collimator size, the median V12 and V10 values for the brainstem and D0.03cc of the ipsilateral temporal lobe were within safe limits, which suggests that ZAP-X GRS delivers radiation to the target with a rapid fall-off, minimizing collateral damage to surrounding tissues. The aim of future studies will be to compare clinical and dosimetric results with the use of the 4-mm collimator. The study conducted by Paddick et al. (2023) provides a thorough comparison of contemporary SRS platforms, benchmarking them against earlier iterations of the technology. The findings reveal notable advances in the performance of current devices, highlighting improvements in treatment plan quality. Overall, the findings from this study emphasize that the ZAP-X platform is a valuable option in SRS, particularly for cases requiring a steep dose gradient and high precision in targeting lesions close to critical structures [6,15].

Regarding safety, the complication rate was low, with only 16.6% of patients experiencing mild ipsilateral facial hypesthesia. This side effect is consistent with previous studies on SRS for TN and is often transient. Only a small percentage of patients experienced pain recurrence, which is within the expected range for SRS. The use of ZAP-X GRS for second-line treatment after recurrence appears to be a viable option, showing positive outcomes following retreatment [1,4,10-14].

## Conclusions

Overall, the results of this study suggest that ZAP-X GRS is a promising new technology for the treatment of refractory TN, providing high rates of pain relief, minimal complications, and precise dose delivery to the trigeminal nerve. Given the favourable outcomes observed in this cohort, further studies with larger patient populations and longer follow-ups are needed to fully assess the long-term efficacy and safety of this approach. However, these early results are encouraging and support the use of ZAP-X as a safe, effective, and minimally invasive treatment option for patients with refractory trigeminal neuralgia.
